# Preparing healthcare providers to use polygenic risk scores: a qualitative study of learning needs and educational preferences

**DOI:** 10.1136/bmjopen-2025-111898

**Published:** 2026-05-07

**Authors:** Amy Clark, Courtney K. Wallingford, Jennifer Berkman, Aideen McInerney-Leo, Amy Nisselle, Bronwyn Terrill, Nathan Palpant, Mary-Anne Young, Paul A. James, Tatiane Yanes

**Affiliations:** 1 Dermatology Research Centre, Frazer Institute, The University of Queensland, Brisbane, Queensland, Australia; 2 Graduate School of Health, University of Technology Sydney, Sydney, New South Wales, Australia; 3 Murdoch Childrens Research Institute, Parkville, Victoria, Australia; 4 Department of Paediatrics, The University of Melbourne, Parkville, Victoria, Australia; 5 Clinical Translational and Engagement Platform, Garvan Institute of Medical Research, Sydney, South Australia, Australia; 6 Faculty of Medicine and Health, UNSW Medicine & Health, School of Clinical Medicine, St Vincent’s Healthcare Clinical Campus, Sydney, New South Wales, Australia; 7 Parkville Familial Cancer Centre, Peter MacCallum Cancer Centre and the Royal Melbourne Hospital, Parkville, Victoria, Australia; 8 Sir Peter MacCallum Department of Oncology, The University of Melbourne, Parkville, Victoria, Australia

**Keywords:** Genomic Medicine, EDUCATION & TRAINING (see Medical Education & Training), Health Workforce

## Abstract

**Abstract:**

**Objectives:**

Polygenic risk scores are increasingly available to consumers to provide an estimate of the genetic contribution to health conditions. However, healthcare providers report limited knowledge and confidence using polygenic risk scores. Clinical implementation necessitates educational programmes to support clinicians to integrate this new test into practice. This study aimed to identify healthcare providers’ learning needs and preferences for polygenic risk education to inform the design of tailored education initiatives.

**Design, setting and participants:**

This pragmatic qualitative study used focus groups to capture healthcare providers’ perspectives. To ensure informed responses, genetic healthcare providers with prior experience using polygenic risk scores, and/or who had completed polygenic risk education were recruited to participate in focus groups or interviews (n=30). There were no exclusions based on country of practice. Recordings were transcribed and content analysis conducted to identify learning needs. Themes related to education engagement were mapped to the capability, opportunity and motivation model for behaviour change.

**Results:**

Among this cohort of experienced providers, residual gaps existed in polygenic risk-related knowledge, skills and local guidelines to inform practice. Learning needs encompassed: (i) polygenic risk-specific knowledge, and (ii) communication skills needed to discuss results and facilitate risk management. Themes related to engaging with polygenic risk education mapped to capability included awareness of, and access to educational resources and initiatives, including practice resources and position statements from professional bodies. Time-poorness was a primary barrier to accessing education. Opportunities comprised of building on existing workplace training and activities such as multidisciplinary team meetings and journal clubs. All participants noted that motivation for completing polygenic risk training was primarily driven by a desire to improve patient-centred care and clinical outcomes.

**Conclusion:**

This study highlights priority learning areas to inform the development of tailored polygenic risk education initiatives, and resources and delivery strategies that meet the identified needs. Participants’ expert insights reveal potential barriers as well as solutions to engaging healthcare providers with polygenic risk score education to ultimately facilitate implementation into clinical practice.

**STRENGTHS AND LIMITATIONS OF THIS STUDY**

- Use of capability, opportunity and motivation model of behaviour change model, offering theory-driven approach to understanding behavioural influences on engagement in polygenic risk score (PRS) education.
- Focus groups encouraged interactive discussion, helping to generate richer and more nuanced data.
- Expert cohort with experience using PRS, allowing for insights grounded in real-world practice.
- The sample lacked diversity, being mostly female genetic counsellors working in cancer care, which may limit generalisability.
- The exclusion of PRS-naïve professionals means the study may have missed important perspectives from those new to the field.

## Background

 Polygenic risk scores (PRS) are an emerging genomic tool that estimates an individual’s genetic predisposition to common health conditions, based on the combined effect of many (hundreds to millions) of genetic variants. While each variant has a small effect on health risk, their cumulative impact can stratify individuals into different levels of risk across a population.^1^ Unlike traditional genomic testing, which typically applies to a small subset of the population, PRS can be generated for all individuals and for most complex health conditions.^2^ PRS can be considered a patient’s intrinsic background genomic risk and can be used alongside other risk factors to inform personalised risk estimates.^3^ Polygenic risk is increasingly being integrated into clinical practice, with several commercial laboratories now delivering PRS testing. Healthcare providers (HP) recognise the potential clinical utility of PRS and that testing will become integrated into future practice.^3 4^ However, healthcare providers frequently report a lack of knowledge and confidence using PRS.^5^,^11^

The lack of HP education regarding PRS has been identified as a key implementation barrier, including by expert professional organisations.^12^ There are limited PRS educational initiatives for HPs, and the providers’ learning needs and preferences are not well understood. Historically, PRS education for HPs has been aligned with research programmes and delivered limited to participating providers.^8 13^ While these studies increase self-reported knowledge and confidence using PRS, they were also time intensive to deliver, not available beyond the study, and their clinical impact has not been evaluated.^7 14 15^ Thus, novel approaches to PRS education are needed to support scalable delivery methods that consider long-term changes to clinical practice.

Incorporation of stakeholder perspectives early in the development of new education programmes is essential to identify learning needs and facilitators/barriers to programme engagement.^16^ The capability, opportunity and motivation model of behaviour change (COM-B) offers a framework for understanding factors that enable providers to engage with and apply new knowledge.^17^ This model posits that behaviour change (ie, engaging with PRS education) occurs when three conditions, capability, opportunity and motivation, are met. Capability includes having the necessary knowledge/skills (psychological) and physical ability (physical) to conduct the behaviour. Opportunity refers to external factors that make the behaviour possible (physical) and a social environment supportive of the behaviour (social). Lastly, motivation describes planning and decision-making processes (reflective) and development of habits and impulses (automatic). Application of this model enables systematic identification of factors influencing uptake of education, which will strengthen the development of a theory informed strategy for PRS education.

This study aimed to (i) identify GHPs’ learning and resource needs for PRS delivery across clinical disciplines and (ii) identify strategies to best engage clinicians in PRS education. Findings will inform the development of future PRS education initiatives for healthcare providers. The study was approved by the University of Queensland Human Research Ethics Committee (2022/HE001551) and the University of Technology Sydney Human Research Ethics Committee for ratification (ETH22-7717). All participants provided informed consent for the study.

## Methods

### Study design and participant recruitment

This qualitative study used focus groups to identify genetic healthcare providers (GHP) learning needs for PRS and to explore barriers and facilitators to engagement with PRS education initiatives; accordingly, recipients of PRS advice were not included, as the study focused specifically on clinician learning needs and engagement preferences. Eligible participants were GHPs (ie, healthcare providers with genetics training including genetic counsellors, clinical geneticists and laboratory-based staff). GHPs were chosen as the primary participant groups as they are at the forefront of PRS implementation and have a role in supporting education of other healthcare providers.^13 18^ Eligibility was also restricted to providers with prior experience using PRS, including ordering and returning results. This criterion aimed to capture informed opinions from providers who have real-world experience using PRS, and therefore, have insight into the required knowledge and skills for clinical PRS use.

Various strategies were employed to facilitate recruitment internationally. We approached several international professional genetics groups, and information about the study was ultimately distributed via organisations in Australia and the USA (ie, Human Genetics Society of Australasia, the Australasian Society of Genetic Counsellors, the National Society of Genetic Counsellors and the American College of Medical Genetics and Genomics (ACMG)). Information about the study was also posted on social media (ie, X, formerly known as Twitter). Lastly, a list of eligible providers with experience using and/or educated in PRS was compiled by the research team. These providers were invited to participate via email by author AC. To maximise recruitment reach, a snowballing approach was also applied. While this approach can introduce biases in the study population, it can also strengthen recruitment by reaching individuals who might not have otherwise participated. Given the initial low participation rate among clinical geneticists, targeted recruitment was conducted for this population, with the study invitation distributed via email to identified experts in the field and the Australasian Association of Clinical Geneticists virtual newsletter.

Individuals interested in participating were provided with a link to the online consent form and a prestudy survey to capture basic demographic information, eligibility (ie, prior experience/education with PRS) and availability for participating in a focus group. Focus groups were the primary data collection method; however, given scheduling challenges associated with clinical workloads and participation across multiple time zones, individual interviews were offered when attendance at a focus group was not feasible. This approach was adopted to maximise participation and avoid exclusion of eligible participants. Both focus groups and interviews used the same semistructured guide and were analysed using a consistent analytic framework to ensure comparability of data across methods.

### Data collection

An interview guide was developed by the research team encompassing experiences with previous PRS education, learning and resources needs, and strategies to support engagement in PRS education (online supplemental file 1). The COM-B model of behaviour change^19^ was used to inform questions around supporting engagement with PRS education. The interview guide was piloted with four providers in a focus group. All interviews and focus groups were conducted online by female authors AC, a Masters of Genetic Counselling student and educator accompanied by JB, senior genetic counsellor and Doctor of Philosophy (PhD) candidate. Previously described methodology for focus group facilitation was applied.^20^ All focus groups and interviews were recorded, transcribed verbatim and de-identified. AC maintained a reflexive journal after each focus group or interview. Participants were provided with a $25 (AUD) Amazon voucher as an acknowledgement of their time. Data collection occurred between February 2023 and April 2024.

### Data analysis

Content analysis was conducted using NVivo 14 software.^21 22^ First, three focus group transcripts were analysed and units of meaning were assigned to participant answers. These units of meaning were then organised into learning/resource needs or into one of the six subcomponents of the COM-B model. Analysis focused on interpretation of meaning rather than enumeration of responses, as frequency counts do not necessarily reflect conceptual importance in qualitative content analysis.^23^ AC discussed the initial units of meaning and coding tree with JB, TY and CW and adjustments were made on discussion. All transcripts were then coded by AC, and during the analysis period, authors AC, JB, TY and CW frequently met to discuss findings and reconcile any discrepancies in the coding process. Author JB independently re-coded 20% of the data to ensure accuracy of coding, achieving a high inter-rater reliability (Kappa 80%).^24^ Illustrative quotes were selected to reflect key concepts, and findings used to identify strategies to support PRS education and subsequent use in clinical practice.

### Patient and public involvement

No patients or members of the public were involved in the design, conduct, reporting or dissemination plans of this research.

## Results

### Demographics

Of the 65 GHPs who expressed interest in the study, 12 were ineligible due to no prior experience with PRS. Of the 53 eligible individuals, 30 participated in the study (n=21 focus groups and n=9 interviews). Reasons for non-participation included no response to invitation to attend focus group (n=9); incorrect or no contact details provided (n=11); and inability to co-ordinate a mutually convenient time (n=3). Focus groups ranged from 65 to 77 minutes, and interviews from 32 to 48 min. Most participants were females (n=25, 83%), genetic counsellors (n=25, 83%) and/or from Australia (n=26, 87%), with prior PRS experience in the breast cancer setting (n=14, 47%). Mean age was 40 years (range 24–67 years), and there was similar distribution across years of practice and time working with PRS. Participants had prior education or experience with PRS through research studies (n=21, 70%), including Polygenic risk modification (PRiMo)^25^ and Electronic Medical Records and Genomics Network (eMERGE),^26^ or had attended PRS conference presentations or one-off webinars (n=6, 20%). Three participants (n=10%) reported not having completed any PRS education. Table 1 summarises participant demographics.

**Table 1:** Participant demographic characteristics and prior experience using PRS (n=30)

Characteristic	N	%
Gender identity		
Women	25	83
Men	5	17
Age		
20–30	6	20
30–40	9	30
40–50	7	23
50–60	4	13
>60	4	13
Country of practice		
Australia	26	87
USA	4	13
Profession		
Genetic counsellor	25	83
Clinician geneticist	5	17
Years of practice		
0–5 years	9	30
6–10 years	8	27
11–15 years	4	13
>15 years	9	30
Time working with PRS		
<6 months	11	37
6–12 months	7	23
1–2 years	5	17
>2 years	7	23
Had experience returning PRS results for		
Breast, ovarian or prostate cancer	14	47
Prostate cancer	1	3
Conditions other than cancer	5	17
Direct to consumer tests	1	3
Multiple conditions	4	13
Experience with consenting patients only	5	17
PRS training		
As part of a research study	21	70
Conference or webinar presentation	6	20
None	3	10

PRS, polygenic risk scores.

### Learning needs

Participants frequently recognised a training gap for GHPs regarding PRS and screening tests. They acknowledge that current clinical genetic or genetic counselling training focuses on diagnostic genomic testing, which differs from the epidemiological concepts used in PRS and population risk stratification. Overall, there was a consensus that providers should understand the concept of genome-wide association studies (GWAS), how GWAS are used to inform PRS development, integrated risk and common epidemiological methods of evaluating risk assessment models. Additional PRS considerations included understanding clinical context/setting for test use, impact of ancestry on result interpretation and confidence in the score (eg, arising from missing Single nucleotide polymorphisms [SNPs]). Regarding clinical use, participants frequently recognised the strong evidence for clinical validity, modelling data supporting PRS and potential clinical applications. They were aware that current clinical trials are still building evidence for real-world clinical utility, which limits the availability of clinical guidelines specific to PRS and integrated risk. The lack of clinical guidelines was viewed by many as a primary barrier for testing uptake, prompting participants to note that any future PRS education will have limited value without guidelines to inform subsequent patient risk management.

There were varying views about the depth of knowledge regarding the statistical and epidemiological processes required to use PRS in clinical practice. Most participants felt that only a gist understanding of these processes was needed to interpret reports, identify potential issues, communicate with laboratories and deliver results to patients. Conversely, a subset of participants wanted extensive background information on PRS development, statistical modelling and evaluation. For these individuals, having an in-depth understanding of the technical aspects of PRS was seen as enhancing their capacity to interpret and communicate information to patients. Regardless of the level of background knowledge, participants overwhelmingly agreed that PRS education should have a strong focus on direct clinical implications for patients and a practical breakdown of tasks such as report/result interpretation and risk communication.

Nearly all participants, particularly genetic counsellors, felt that GHPs already had the necessary skills to communicate complex risk information needed for PRS. These skills included capacity to distill complex information to a level that is appropriate to the patient, active listening, reflective practice and exploring the psychological and familial implications of results, which are all transferable to the PRS setting. However, a need for greater education and resources to support communicating integrated risk (ie, risk scores where PRS, monogenic and other non-genetic risk factors are combined) was noted. Specifically, participants raised examples of difficulties they encountered attempting to communicate both Mendelian and polygenic risk inheritance, as well as providing multiple risk figures (ie, 5-year, 10-year and lifetime). Participants suggested practice-based examples and resources would be helpful to support PRS communication, specifically, sample explanations for patients, analogies, common patient questions and role-play videos. Lastly, several participants acknowledged the influence of patient behaviour (eg, lifestyle factors) on integrated risk scores and identified it as an opportunity to support patients to practice favourable and/or preventative health behaviours. However, participants did not feel sufficiently trained in behaviour change methodologies to support patients in this area. Table 2 summarises participant learning needs using illustrative quotes.

**Table 2:** Summary of genetic healthcare providers’ learning needs related to use

Theme	Quote
PRS-specific knowledge base	
Not standard training for GHPs	“I trained over a decade ago, and we unfortunately had very little true formal GWAS training…there’s very little of that training or education for genetic counsellors. Because we generally haven’t had to use or decide to use screening tests…We’ve ordered diagnostic gene tests and let the clinicians handle the mammograms and the cholesterol panels…” (Participant 24, Genetic Counsellor)
Fundamental knowledge: GWAS, PRS development, evaluation and integrated risk	“I like understanding the first principles or the kind of underlying basis of whatever’s sort of being calculated. Like not the, not an enormous detail, but just enough so that you kind of have a rough idea about how these things are calculated.” (Participant 2, Clinical Geneticist) “I think there needs to be some foundational education about things like model discrimination, internal and external validation” (Participant 24, Genetic Counsellor)
PRS considerations	“I guess the question that the problem needs to be asked with an educational point of view, well what’s it being used for?” (Participant 27, Clinical Geneticist) “…how we use the polygenic risk in terms of the different ethnicities and how we deal with that and how I discuss that with the patient”. (Participant 1, Genetic Counsellor) “…having an understanding of whether or not that (missing SNPs) will actually influence the accuracy of the risk assessment, I think is really important…I’d sort of assume to read in these reports…*”* (Participant 17, Genetic Counsellor)
Emerging evidence	“I have the impression…we are kind of at that we have clinical validity, but not necessarily (clinical) utility and that’s kind of what we’re figuring out, isn’t it?” (Participant 2, Genetic Counsellor)
Clinical guidelines	“Without guidelines, it’s hard to convince people that this is something they need to do.” (Participant 27, Clinical Geneticist) “Do I put this woman on insulin because she’s freaking out about her slight increased risk of diabetes? And that’s a really good question. What needs to be done? What criteria, what guidelines?” (Participant 20, Genetic Counsellor)
Level of knowledge required	“I sit somewhere in between wanting to know absolutely everything and absolutely nothing about a PRS. There are some technical details that I need to know when I’m giving a result. If something doesn’t make sense to me on a report, I will go to the lab and say, you need to explain this because it doesn’t make sense why there looks like they’ve got a really high PRS, you’ve reported them as moderate…and they help explain that. But do I ever really want to do my own PRS score? Probably not.” (Participant 28, Genetic Counsellor) “If I can understand how something is generated, then it helps me to be able to communicate it.” (Participant 8, Genetic Counsellor) “There will be a kind of subgroup of patients who are really quite interested in how a PRS is calculated and having that knowledge up my sleeve will be useful*.*” (Participant 16, Genetic Counsellor)
Direct clinical implications	“What I really gained from doing the PRiMo (PRS) training…was a really kind of practical breakdown of, well, if this is, this is the information we’re gonna provide you on a report and then being able to translate that into useful clinical relevant information that the patients can then use to inform their risk management, or risk perception.” (Participant 13, Genetic Counsellor)
Communication skills	
GHP are already trained in the skills needed for PRS communication	“You need to be able to sense the level of your client’s understanding. You need to be able to work out what language to use with them. You need to be able to use those active listening skills and reflective statements to try and figure out, are they with me? They’re all the same skills that you use, no matter what you’re counselling about.” (Participant 19, Genetic Counsellor)
Complex inheritance discussion	“It can become quite a complex discussion and that is quite difficult when someone has inherited the familial mutation. So, you’ve given them a result that has huge emotional implications and then trying to discuss something quite complex with them.” (Participant 3, Genetic Counsellor)
Practice-based examples and resources to support PRS communication	“We all pick up useful analogies, or ways of phrasing things as we go through our training, and if we can concentrate that into a (PRS) training course, so that we don’t need to observe other people doing it, that’d be amazing.” (Participant 2, Clinical Geneticist) “…experiential learning is my better preferred model and if I’ve had the chance to role play something a bit new as opposed to watching a role play, I’m probably more likely to be able to try it out … until I actually have to do it, that’s not putting me on the spot and seeing if I’m fumbling over my words or I really don’t understand this.” (Participant 26, Genetic Counsellor)
Potential for behaviour change	“I’m really interested in the combined (score) because it seems to be a little bit more powerful. It also has the potential for people to see where the risk factors are and how to change them and it gives them back power around some of these conditions.” (Participant 28, Genetic Counsellor)
GHP do not feel equipped to influence behaviour change	“It’s (behaviour change) is not something I ever had experience in communicating. It’s not something that was touched on in my training at all. So, it was entirely new language phrasing.” (Participant 14, Genetic Counsellor)
	“… my ability to change someone’s entire behaviour with a one-off 20-minute conversation is very minimal.” (Participant 14, Genetic Counsellor)

GWAS, genome-wide association studies; PRS, polygenic risk scores.

### Factors influencing engagement with PRS education

This study used the COM-B model of behaviour change to comprehensively map the factors impacting participants engagement with PRS education.

#### Capability

Two overarching concepts were identified relevant to psychological capability: (i) customised learning depending on the individual’s knowledge base and (ii) dissemination and awareness of educational resources. When reflecting on prior professional development and education opportunities, many participants noted limited consideration of varied knowledge levels and lack of catering for different learning styles, which negatively impacted subsequent engagement. Lack of awareness of existing educational resources was also noted as a barrier to engagement in PRS learning activities. For example, some participants were unaware of resources developed by professional bodies that outline considerations for clinical implementation of PRS, such as NSGC Practice Resource on use of PRS.^27^ Participants suggested that collating PRS resources into a central PRS learning hub may facilitate access and improve dissemination of resources. Novel approaches to enhance dissemination were described including creating PRS special issues or dedicated sections within professional body newsletters and ensuring newly developed online resources are easily identified by online search engines.

Participants identified barriers to physical capability impacting their engagement with PRS education, including (i) accessibility issues and (ii) being time poor. Some participants discussed attempts to access PRS training modules linked to research programmes, with access blocked or requiring special permission. Participants suggested flexible delivery modes, such as dividing online content into small micro-lessons that could be accessed over time. However, they also recognised the potential limitations of web-based programmes, such as higher levels of non-completion/disengagement due to information density and distraction.

#### Opportunity

Participants described potential enhancements to physical opportunities that included (i) building PRS education into existing workplace training and (ii) facilitating opportunities to discuss cases with peers and experts. Adapting current continuing professional development (CPD) activities to incorporate PRS, rather than additional programmes, was highly valued by participants. Clinical supervision sessions, multidisciplinary team meetings and journal clubs were identified as potential settings in which PRS education could be incorporated. Additionally, GHPs strongly valued access to experts within multidisciplinary teams, journal clubs or within research study teams. This approach was seen as enhancing peer-to-peer discussions, learning opportunities and the development of PRS-related skills. Lastly, based on the value of peer-to-peer learning, participants suggested developing forums for PRS interpretation. Specifically, participants outlined that providers could submit complex PRS cases and receive expert feedback to help manage their own cases, as well as facilitate reciprocal learning through case discussions.

Factors impacting social opportunities to engage in PRS education were encompassed by two main themes: (i) workplace culture/values toward PRS education, and (ii) institutional support. Participants highlighted that engagement in PRS education would be enhanced through a positive workplace culture (including peer attitudes) that values professional development. Participants who had been involved in PRS implementation studies stated that their workplaces had strong cultures of collective commitment to clinical research and building evidence-based practice, which facilitated the opportunity to engage in training. Specifically, participants outlined important workplace values that enhanced participation in PRS training including collective decision-making around trial participation, inclusive team training to ensure comfort and standardisation, and prioritisation of time for training. GHPs acknowledged that this team setting, and collective culture would be a challenge for those working in smaller teams or remote settings. Participants also pointed to a lack of institutional support (ie, protected time on an institutional level and covered costs to train staff) as a major barrier to PRS education engagement. One suggestion to secure more support on an institutional level was to align the learning outcomes of local education initiatives with strategic priorities of the organisation, where feasible. While this idea was not frequently raised, it generated much interest and discussion during focus groups. Finally, some participants noted concerns around the additional clinical consultation time thought to be necessary to implement PRS into clinical practice.

#### Motivation

Automatic motivation to engage in PRS education was primarily driven by a desire to improve patient-centred care. Specifically, nearly all GHPs felt that PRS could enhance personalised care, provide answers for families about unexplained heritability (ie, when no causal pathogenic variant is identified despite a significant family history), and/or provide more accurate risk assessments. Motivation to engage in PRS use was further driven by past experiences, with some GHPs describing increasing referrals related to PRS. Thus, providers wanted to engage with PRS education to build their confidence and capabilities in applying PRS in clinical practice. Participants suggested that their confidence and capabilities could be best enhanced through practice-based training, which could include discussions, practice role plays and feedback with peers.

Participants identified factors that influenced their reflective motivation, namely: (i) upskilling in PRS as a professional standard and (ii) wanting to facilitate consistent care for families. GHPs wanted to maintain professional standards and stated that they “do not want to be left behind” in what was described as the next frontier in genomic medicine. In some cases, participants felt that having skills with PRS communication would improve their employability. Participants suggested attaching CPD points to educational activities in recognition of the time taken to engage with PRS education but noted that its impact may be limited due to the volume of other available CPD activities. Lastly, participants wanted to ensure consistent case management when using PRS in clinical practice and suggested this could be best facilitated through practice-based and group training. Table 3 summarises the identified factors influencing PRS education engagement.

**Table 3:** Capabilities, opportunities and motivations that facilitate clinicians building their PRS knowledge and skills

Theme description	Representative quote	Implication for training
Capability: psychological (knowledge and mental capacity)		
Building on existing knowledge base	“It’s one of the real challenges…of most workshops that I’ve been to where people just haven’t been able to accommodate that diversity of expertise.” (Participant 2, Clinical Geneticist)	Customisable education to accommodate varied baseline knowledge levels and learning needs.
Awareness of existing education	“I don’t know if that resource has been integrated into our training curriculum yet. I think it’s listed…as a supplemental resource that students are encouraged to check out…But honestly, it sounds bad, but I feel like nobody else knows about the (NSGC) practice resource*”* (Participant 24, Genetic Counsellor)	Dissemination and visibility of resources to improve knowledge of training resources Development of central PRS resource hub.
Capability: physical (physical capacity)		
Accessibility—ability to attend or access existing education programmes/initiatives.	“it’s really hard for me to attend an actual place to do training…(anything) that makes things way more accessible for me whenever via, you know, telehealth or virtual or something I can do online in my own time.” (Participant 18, Genetic Counsellor)	Ensuring accessible education
Time poorness—finding time to complete education.	“…having smaller modules is really good because, you know, we’re really busy. Sometimes you’ve got a spare half hour or a spare hour and being able to just break it down and do a small little component one day and then maybe another hour another day makes it a little bit more feasible.” (Participant 8, Genetic Counsellor)	Micro learning, chunking information.
Opportunities: physical (inanimate/physical environment)		
Integration of PRS education in current workplace education	“We’ve ended up talking about it in a couple of our supervision sessions. That’s actually been really helpful to really just like, throw it out there. These are the experiences, these are the challenges we have, and getting that group feedback and ideas, has been really supportive.” (Participant 14, Genetic Counsellor)	Embedding PRS education into MDTs, journal clubs, supervision and grand rounds.
Education initiatives that facilitate expert and peer case discussion	“…the national variant review meeting…people bring their cases. All the labs are there, all the services are there. You work through these tough variants, and you come away with something and it’s like actually super worthwhile.” (Participant 3, Genetic Counsellor)	Online case discussion meetings with subject matter experts.
Opportunities: social (social environment/culture)		
Workplace culture that values PRS education	“Our unit did it as a group. So that made it very easy… it’s actually something that’s recommended by your workplace… you’re doing it with people, you know and feel comfortable asking questions.” (Participant 1, Genetic Counsellor)	Demonstration of importance through peer promotion (eg, inclusion of PRS-focused sessions in conferences).
Institutional support for PRS education	“I think that’s where we struggle the most. It always ends up being something that we tack on or squeeze in…unless we have a, a way of actually future proofing it or planning it.” (Participant 8, Genetic Counsellor)	Identifying how PRS training aligns to health system policies and strategic plans. Blocked time, funded education, workload considerations, upskilling focus aligned with strategic priorities.
Motivation: automatic (habits and impulses)		
Consistently encountering PRS results or referrals drives a need to upskill	“At the beginning it kind of fell in my lap… I ended up seeing a bunch of referrals for that reason. So, I started to educate myself, just trying to figure out what to tell the patients in that space.” (Participant 23, Genetic Counsellor)	Identify examples of referral/case scenarios in educational resources (ie, case-based learning) Consider patient needs, eg, frequently asked questions by patients
Desire to provide personalised care for patients	“…once you look at (lifestyle) factors and someone realises that they have control over some of those factors, it’s a game changer…They feel like there’s real, power that’s been given to them to know this information and for some reason it is way less scary than doing germline…PRS feels like it’s much more in control.*”* (Participant 28, Genetic Counsellor)	Highlight individual clinical and personal utility of PRS in educational programme.
Motivation: reflexive (conscious thought and planning)		
Facilitating consistent care across services	“… I don’t know what we would do with if this becomes mainstream clinical… what we do with families with moderate risk gene…And then when we start getting families with different risk information for different family members…from an industry perspective, some sort of agreement about how we’re going to deal with that when it comes.” (Participant 7, Genetic Counsellor)	Develop PRS model of care alongside educational programmes to ensure consistent evidence-based practice.
Maintaining professional standards	“It was the feeling that PGS was going to be inevitable, and I better learn about it now or I’ll be left behind.” (Participant 3, Genetic Counsellor)	Recognising PRS education contributes to CPD and professional standing

CPD, continuing professional development; MDT, multidisciplinary team; PRS, polygenic risk scores.

## Discussion

This study explored learning needs and factors influencing HP engagement in PRS education. Preference for patient-centred PRS education was a key finding, with providers emphasising risk communication and clinical management over comprehensive knowledge of PRS development and evaluation. Overall, participants favoured customisable, case-based and practice-based learning, as well as written materials that included exemplary explanations and responses to common patient questions. Based on study findings, specific suggestions for future PRS education were proposed that included resource differentiation to accommodate varied learning needs, flexible modules and accessibility through development of a central PRS resource hub. Time constraints, competing workloads and lack of systemic support were major barriers to completing PRS education. Integrating PRS education into workplace training, such as journal clubs, case discussions and identifying peer champions were suggested avenues to address these barriers.

PRS represents a new landscape in healthcare provider education and practice. Understanding PRS development requires analytical and statistical skills, such as interpreting risk estimates, evaluating model performance and understanding the implications of population genetics.^28^ These skills are not routinely included in standard medical education, nor is it considered in practice training competencies for GHPs in Australia or the USA.^10 11^ Consequently, providers lack formal training in these areas, highlighting a critical gap in preparing the workforce for the integration of PRS into clinical care. The lack of PRS-specific clinical guidelines was described as a key barrier to more widespread test implementation.^7^ Clinical guidelines were described as necessary to support providers in delivering healthcare recommendations post-testing. Providers recognised that there has not been sufficient time to generate long-term evidence for clinical utility that can be used to inform clinical guidelines.^29^ This evidence gap presents a significant challenge for clinical education, as providers have limited access to clear information about clinical and lifestyle/behavioural interventions that can be offered to patients receiving PRS results. Thus, developing PRS-specific clinical guidelines should be an area of priority for future research.

Participants noted that their current skillset in communicating complex risk information was transferable to the PRS setting. However, skill gaps were identified in the communication of integrated risk information. Participants also identified challenges in delivering different risk time frames, such as 5-year, 10-year and lifetime risk figures. Given preferences for patient-centred education, future PRS education development should prioritise point-of-care resources, with visual aids and patient-friendly reports to facilitate risk communication. While visual aids, such as ‘the jar model’,^30^ have been adapted to include monogenic and polygenic testing, assessment of their utility has been more limited beyond psychiatric genetics.^31^ Future research should aim to explore how other existing resources can be adapted to other PRS settings such as familial cancer and cardiac genetics. Furthermore, educational resources could include examples of metaphors and analogies, communication tools which are known to aid communication of complex genetic risk information.^32^

Identification of facilitators and barriers to engagement in clinical PRS education allows for the development of wholistic clinical education strategies. To optimise engagement in PRS education initiatives, HPs expressed a need for customisable and flexible education, with a preference for practice-based learning, consistent with other genomics education studies.^33^,^35^ Practice-based learning aligns with adult learning theory, which highlights the involvement of the participant in planning and evaluation of their learning experience, immediacy and problem-based learning as integral to engagement.^36^ These education strategies are associated with improved clinician performance and patient outcomes.^37 38^ Furthermore, participants noted limited time, competing workloads, lack of institutional support and lack of awareness of existing education resources as barriers for PRS education engagement. These barriers are well recognised in the literature^39 40^ and suggest PRS education programmes should consider implementation strategies that foster institutional/workplace support^39 40^ and broaden reach through centrally located education initiatives (ie, a designated PRS education website).^26^

Implementing these strategies cannot rely on the individual clinician. Systemic challenges in genomics education are well recognised and are not unique to PRS. Although models of delivery vary across health systems, best-practice approaches support shared responsibility between healthcare organisations, professional training bodies and clinical genetics services.^16^ Organisational policies and system-level structures are therefore needed to support training and evaluation of clinicians as genomic technologies such as PRS are implemented.^16 41^ Furthermore, the cost and workforce implications of genomics education models remain poorly characterised. While experiential approaches are effective but resource-intensive, scalable strategies such as blended learning and genomic champions may offer more sustainable alternatives.^42^ Future research should evaluate the comparative cost, workforce and system-integration implications of PRS education models to inform long-term implementation.

This study included a participant cohort of predominantly female genetic counsellors from Australia, with experience in the cancer setting, which is representative of the Australasian GHP profile of which two-thirds are female genetic counsellors with a median age of 37.^43^ The use of expert providers captured learning needs and education engagement that was informed by real world practice. Nevertheless, naïve users and those from other specialties may have additional learning needs not captured in this study. A further methodological limitation is that formal member checking was not undertaken; however, analytical rigour was supported through iterative team-based analysis and the use of a theory-informed framework. While several attempts were also made to increase participant representation internationally, representation remained low beyond Australian participants. Future studies should aim to explore the views of PRS-naïve users and other healthcare professional groups, particularly in more diverse international settings. This study also did not capture patient perspectives. While there is emerging evidence that PRS is generally associated with minimal psychological distress, some individuals have indicated confusion regarding result interpretation. ^44^Patient perspectives should be incorporated into the development of future PRS education initiatives to ensure end products are person-centred and address the need of the population in which they serve. While the range of international participants remained small, the use of the COM-B model strengthens the study by providing a theory-informed approach to data analysis and interpretation and increasing the generalisability of our findings. Additionally, data collection via focus groups allowed dynamic group discussion, where participants built on each other’s ideas to generate deeper and more comprehensive exploration of topics.

This study identifies key learning needs and engagement factors for polygenic risk score education among genetic healthcare providers, with implications for the broader healthcare workforce. Key educational priorities include foundational knowledge of PRS development and integrated risk communication with clear clinical relevance. The development of clinical guidelines and standardised care models is also needed to support risk management following PRS and integrated risk assessment. To enhance provider engagement, PRS education and training should be customisable, flexible and accessible, preferably integrated into existing workplace-based training and delivered in team settings with employer support. When designed with these principles from the outset, PRS education and training led by the genetic healthcare workforce can provide a scalable foundation for educating the broader healthcare workforce.

## Supplementary material

10.1136/bmjopen-2025-111898 online supplemental file 1

10.1136/bmjopen-2025-111898 online supplemental file 2

## Data Availability

Data are available upon reasonable request.
