# Supplementary material for: Preparing healthcare providers to use polygenic risk scores: a qualitative study of learning needs and educational preferences
Source: BMJ Open. 2026 May 7;16(5):e111898. doi: 10.1136/bmjopen-2025-111898 (PMC13157732; doi:10.1136/bmjopen-2025-111898)
Supplement: Supplementary file 3 [file bmjopen-2025-111898.reviewer_comments.pdf]

PEER REVIEW HISTORY

BMJ Open publishes all reviews undertaken for accepted manuscripts. Reviewers are asked to complete a checklist review form and are provided with free text boxes to elaborate on their assessment. These free text comments are reproduced below.

ARTICLE DETAILS

Title (Provisional)

Preparing healthcare providers to use polygenic risk scores: a qualitative study of learning needs and educational preferences

Authors

Clark, Amy; Wallingford, Courtney K; Berkman, Jennifer; McInerney-Leo, Aideen M; Nisselle, Amy; Terrill, Bronwyn; Palpant, Nathan; Young, Mary-Anne; James, Paul A.; Yanes, Tatiane

---

VERSION 1 - REVIEW

Reviewer 1

Name Argent, Andrew

Affiliation Red Cross War Memorial Childrens Hospital, Paediatric Intensive Care Unit

Date 29-Nov-2025

COI None

General comments

The study used focus groups with genetics healthcare providers with experience and training in the use of polygenic risk scores to highlight leanring needs for potential uses of PRS in the clinical setting.

The majority of participants were from Australia or relatively affluent countries.

It is striking that the use of polygenic risk scores is relatively recent, with the implication that many healthcare providers who are providing and using polygenic risk scores will not have been exposed to this modality during their original training. That means that this addresses the problem of how one ensures that healthcare practitioners are kept up to date with new developments during their careers.

The study is interesting and addresses important issues.

Specific comments

Title

I believe that it is inappropriate to use the abbreviation polygenic Risk scores (PRS) in the title, and suggest that the term is put in full in the title.

Given that the vast majority of participants were from Australia, it may not be appropriate to entitle this study “international”

Introduction

The introduction provides a clear overview of the subject, and the need for this particular investigation.

Methods

Study design and participant recruitment

It is unfortunate that only providers were included (not recipients of advice on the basis of PRS).

Was any attempt made to check with the participants that they agreed with the themes and issues arrived at by the researchers?

Results

Demographics

Do the authors have a sense of why there was such a high dropout from people interested in the study through to those who actually participated?

Do the authors have any sense of how the demographics of the participants compares to the demographics of healthcare providers working in this field?

Learning needs

It is interesting that there was a difference of opinion between participants about the need for knowledge about underlying principles of statistics and epidemiological processes in PRS utilization.

It would have interesting to have a little more information about where the healthcare practitioners derived most their clinical training in the area of PRS applications.

Discussion

The study raises a number of complex issues including: who should carry responsibility for the development of educational programmes when new techniques are devised and implemented; what is the role of employers / healthcare organizations in ensuring that practitioners are adequately trained in new techniques and systems; how should the standard of care / performance of healthcare workers be evaluated – particularly when implementing new concepts and processes?

It also be very interesting to have a sense of the cost and personnel implications of different training models and systems to ensure that practitioners are up to date.

**Reviewer**	**2**
**Name**	**Fatum, Muhammad**
**Affiliation**	**University of Oxford, IRS**
**Date**	**16-Jan-2026**
**COI**	**None**

This study aims to identify healthcare providers (HPs) learning and resource needs and preferences for PRS- (Polygenic risk scores) education to inform the design of tailored education strategies and initiatives. Genetic Healthcare Providers (GHPs) with prior experience/education using PRS were recruited to participate in focus groups or interviews (n=30). Recordings were transcribed and content analysis conducted to identify learning needs. Themes related to education engagement were mapped to the Capability, Opportunity and Motivation model for Behavior change (COM-B). The study has been granted ethical approval.

The study is a qualitative one that highlights priority learning areas and the HPs insights on potential barriers as well as the suggestion of solutions to facilitate the implementation of PRS into clinical practice.

Themes related to capability included awareness of, access to educational resources and initiatives, including practice resources and position statements from professional bodies. Time-poorness was a primary barrier to accessing education.

Opportunities comprised of building on existing workplace training and activities such as multidisciplinary team meetings and journal clubs. All participants noted that motivation for completing PRS training was primarily driven by a desire to improve clinical patient-centred care .

This study deals with an emerging and increasingly important and clinically pertinent topic and offers COM-B model to understand behavioral influences on engagement in PRS education. Introduction presents very well the clinical background, its pertinence and the aims of the study.

Methods: snowballing recruitment approach employed can cause a bias in the study's participants selection, although it may have been necessary to recruit enough participants. Also, participant input was sought in two different methodologies with either focus groups or interviews to accommodate for scheduling challenges. This needs to be discussed further in methods and/or the discussion section, to clarify how this bias was minimized as a result of using those two different methodologies.

Results: General presentation of the learning needs results, capabilities, opportunities and motivation (varying views, most participants overwhelmingly agreed, many/some participants etc) without providing figures and percentage of participants that agreed on a need. The authors should semi/quantify these findings (figures/percentages of participants agreeing to the different pointers) and including them in the text and/or tables ie table 2 and table 3

Discussion is comprehensive and summarizes and discusses the different findings and satisfactorily relates to the study's strengths and weaknesses. The study findings and conclusions relate more to genetic HPs so would be reasonable to tighten the scope of the generalization/conclusions to genetic/genomic PRS, throughout the different sections of the manuscript.

In summary. A well designed and clearly written study with sound methodology that offers important engagement themes using the COM-B behavior change framework. The results section with the related tables need to add semi/quantitative presentation of the different COM-B related needs/facilitators findings and the related intervention suggestions, to grant more accuracy and validity to the findings and the derived recommendations.

VERSION 1 - AUTHOR RESPONSE

Reviewer 1

Reviewer comment	Response and changes made
**Title:**<br>The abbreviation PRS should not be used in the title.	PRS has been changed to 'polygenic risk scores' in the title.
The term "international" may not be appropriate given participant demographics.	We agree with the reviewer and have removed the word 'international' from the title. We have noted this limitation in the Discussion, page 23 lines 387-391:<br><br>"While several attempts were also made to increase participant representation internationally, representation remained low beyond Australian participants. Future studies should aim to explore the views of PRS-naïve users and other healthcare professional groups, particularly in more diverse international settings."
**Methods:**<br>It is unfortunate that only providers were included (not recipients of advice on the basis of PRS).	We agree this is important to consider patients and consumers perspectives of PRS. However, these perspectives were beyond the scope of the study, which focused on healthcare providers. Additionally, there have been several studies capturing patient experience with PRS from where we can infer recipients' learning needs. Based on our interpretation of current research, a more gap clearer gap in the literature was refining healthcare providers PRS learning needs.

	We have clarified the study scope of the study in the Methods, page 5, lines 107-110:<br><br>"This qualitative study used focus groups to identify genetic healthcare providers' learning needs for PRS and to explore barriers and facilitators to engagement with PRS education initiatives; accordingly, recipients of PRS advice were not included, as the study focused specifically on clinician learning needs and engagement preferences."<br><br>We have also noted this limitation in the Discussion, page 23 lines 389:<br><br>"This study also did not capture patient perspectives. While there is emerging evidence that PRS is generally associated with minimal psychological distress, some individuals have indicated confusion regarding result interpretation[48].Patient perspectives should be incorporated into the development of future PRS education initiatives to ensure end products are person-centred and address the need of the population in which they serve."
Was any attempt made to check with the participants that they agreed with the themes and issues arrived at by the researchers?	We have clarified that formal member checking was not undertaken and have discussed this as a methodological limitation in the Discussion page 23, lines 385-387:<br><br>"A further methodological limitation is that formal member checking was not undertaken; however, analytic rigour was supported through iterative team-based analysis and the use of a theory-informed framework."
**Results:**<br>Do the authors have a sense of why there was such a high dropout from people interested in the study through to those who actually participated?	Our participation rate was 53% (30/53), which we believe is typically high for this population of healthcare providers. The available information for non-participation is reported in the manuscript (see Methods, page 8, line 171-174; see below). No further information was collected, and therefore, we cannot infer further why individuals did not participate.<br><br>"Of the 53 eligible individuals, 30 participated in the study (n=21 focus groups, and n=9 interviews). Reasons for non-participation included no response to invitation to attend focus group (n=9); incorrect or no contact details provided (n=11); and inability to co-ordinate a mutually convenient time (n=3)."
Do the authors have any sense of how the demographics of the participants compares to the demographics of healthcare providers working in this field?	We have added more detail to this comparison to the Discussion, page 23, lines 380-382:<br><br>"This study included a participant cohort of predominantly female genetic counsellors from Australia, with experience in the cancer setting, which is representative of Australasian GHP profile of which two-thirds are female genetic counsellors with a median age of 37.[46]"
It is interesting that there was a difference of opinion between participants about the	Page 10 in line 201 clarifies that this was difference was about the **depth** of knowledge required, not whether this knowledge was required. All participants agreed some level of knowledge of underlying PRS principles were needed:

need for knowledge about underlying principles of statistics and epidemiological processes in PRS utilization	"There were varying views about the depth of knowledge regarding the statistical and epidemiological processes required to use PRS in clinical practice. Most participants felt that only a gist understanding of these processes was needed to interpret reports, identify potential issues, communicate with laboratories and deliver results to patients. Conversely, a subset of participants wanted extensive background information on PRS development, statistical modelling and evaluation."
It would have interesting to have a little more information about where the healthcare practitioners derived most their clinical training in the area of PRS applications.	Additional detail about PRS clinician training has been added in the text. Results, page 8. lines 178-182:<br><br>"Participants had prior education or experience with PRS through research studies (n=21, 70%), including PRiMo [25] and eMERGE[26], had attended PRS conference presentations or one-off webinars (n=6, 20%). Three participants (n=10%) reported not having completed any PRS education."
**Discussion:**<br>The study raises a number of complex issues including: who should carry responsibility for the development of educational programmes when new techniques are devised and implemented; what is the role of employers / healthcare organizations in ensuring that practitioners are adequately trained in new techniques and systems; how should the standard of care / performance of healthcare workers be evaluated – particularly when implementing new concepts and processes?<br><br>It would be interesting to have a sense of the cost and personnel implications of different training models and systems to ensure that practitioners are up to date	We agree with the reviewers' comments. The complexities of genomics education and cost associated have been well documented. We have explored this issue in the discussion. See page 23, lines 367-378:<br><br>"Implementing these strategies cannot rely on the individual clinician. Systemic challenges in genomics education are well recognised and are not unique to PRS. Although models of delivery vary across health systems, best-practice approaches support shared responsibility between healthcare organisations, professional training bodies, and clinical genetics services[17]. Organisational policies and system-level structures are therefore needed to support training and evaluation of clinicians as genomic technologies such as PRS are implemented[44]. Furthermore, the cost and workforce implications of genomics education models remain poorly characterised. While experiential approaches are effective but resource-intensive, scalable strategies such as blended learning and genomic champions may offer more sustainable alternatives [45]. Future research should evaluate the comparative cost, workforce, and system-integration implications of PRS education models to inform long-term implementation."

Reviewer 2

**Reviewer comment**	**Response and changes made**
**Methods:**	We agree that snowballing approach can introduce biases in the study. However, it was a necessary methodological

Snowballing recruitment approach employed can cause a bias in the study's participants selection, although it may have been necessary to recruit enough participants.	approach given the niche area of the study. The snowballing approach allowed participants to share the study invitation with other individuals who met the study criteria and may have missed the study invitation via professional bodies.<br><br>To offset the limitations of snowballing, we included several approaches to recruitment (see Methods, page 6, lines 118-131), including advertising the study in three professional body newsletters and social media. A list of eligible providers was also compiled based on our review of PRS research that had been published in Australia and internationally.<br><br>We have edited the Methods – Study design and participant recruitment to clarify the reasoning for including snowball sampling; page 6 lines 126-128:<br><br>"To maximise recruitment reach, a snowballing approach was also applied. While this approach can introduce biases in the study population, it can also strengthen recruitment by reaching individuals who might not have otherwise participated."
Also, participant input was sought in two different methodologies with either focus groups or interviews to accommodate for scheduling challenges. This needs to be discussed further in methods and/or the discussion section, to clarify how this bias was minimized as a result of using those two different methodologies.	We agree that using two different methodologies can introduce biases. The use of both approaches was discussed in page 6, lines 134-140:<br><br>"Focus groups were the primary data collection method; however, given scheduling challenges associated with clinical workloads and participation across multiple time zones, individual interviews were offered when attendance at a focus group was not feasible. This approach was adopted to maximise participation and avoid exclusion of eligible participants. Both focus groups and interviews used the same semi-structured guide and were analysed using a consistent analytic framework to ensure comparability of data across methods."
**Results:**<br>General presentation of the learning needs results, capabilities, opportunities and motivation (varying views, most participants overwhelmingly agreed, many/some participants etc) without providing figures and percentage of participants that agreed on a need. The authors should semi/quantify these findings (figures/percentages of participants agreeing to the different pointers) and	We acknowledge the suggestion to include semi-quantitative indicators. However, our study used qualitative content analysis to identify and interpret learning needs and engagement factors, rather than to estimate prevalence. We therefore prioritised a qualitative presentation (categories/themes with illustrative quotations) and clarified in the Methods that frequency counts were not reported because counts can imply quantitative generalisability and do not necessarily reflect conceptual importance, page 7, lines 158-160:<br><br>"Analysis focused on interpretation of meaning rather than enumeration of responses, as frequency counts do not necessarily reflect conceptual importance in qualitative content analysis.[24]"

including them in the text and/or tables i.e. table 2 and table 3	
**Discussion:**<br><br>The study findings and conclusions relate more to genetic HPs so would be reasonable to tighten the scope of the generalization/conclusions to genetic/genomic PRS, throughout the different sections of the manuscript.	We agree with this feedback and have adjusted the Conclusion accordingly to reflect that whilst these findings apply to the genetics workforce, this workforce and the findings will ultimately lead education of other HPs and pave the way for greater clinical implementation of PRS. See page 24, lines 400-410:<br><br>"This study identifies key learning needs and engagement factors for PRS education among genetic healthcare providers, with implications for the broader healthcare workforce. Key educational priorities include foundational knowledge of PRS development and integrated risk communication with clear clinical relevance. The development of clinical guidelines and standardised care models is also needed to support risk management following PRS and integrated risk assessment. To enhance provider engagement, PRS education and training should be customisable, flexible, and accessible, preferably integrated into existing workplace-based training and delivered in team settings with employer support. When designed with these principles from the outset, PRS education and training led by the genetic healthcare workforce can provide a scalable foundation for educating the broader healthcare workforce."

VERSION 2 - REVIEW

Reviewer 1

Name Argent, Andrew

Affiliation Red Cross War Memorial Childrens Hospital, Paediatric Intensive Care Unit

Date 11-Mar-2026

COI

Thank you for the responses to the reviewers. I have no further suggestions or comments for this paper.
